# Fludarabine-Cyclophosphamide-Rituximab Treatment in Chronic Lymphocytic Leukemia, Focusing on Long Term Cytopenias Before and After the Era of Targeted Therapies

**DOI:** 10.3389/pore.2021.1609742

**Published:** 2021-04-27

**Authors:** Róbert Szász, Béla Telek, Árpád Illés

**Affiliations:** Division of Hematology, Department of Internal Medicine, Faculty of Medicine, University of Debrecen, Debrecen, Hungary

**Keywords:** treatment, chronic lymphocytic leukemia, fludarabine, cyclophosphamide, rituximab, cytopenia

## Abstract

The widespread application of fludarabine, cyclophosphamide, and rituximab combination is limited due to its toxicity, particularly the prolonged cytopenias. The study aimed to compare the prolonged cytopenias depending on fitness and report real-life data on dose reduction measures and efficacy. According to our database, 120 and 14 patients were treated with FCR between 2011 and 2015 and between 2016 and 2019. Out of the first cohort, 34 patients were treated in subsequent lines. The complete and partial remission rate after first-line treatment was 79%, 16% in the first cohort and 86%, 14% in the second cohort, respectively; and 47%, 35% after non first-line treatment. Based on today’s standards, only 37.5% of the patients were fit for FCR. The frequency of persistent cytopenia was 14%, and it was significantly associated with fitness (*χ* ^2^ (1) = 6.001, *p* = 0.014 for all patients). The small number of FCR treated patients after 2016 shows how the availability of targeted therapies, mostly ibrutinib, in later lines changed the first-line choice. Recently, it is recommended first-line for fit patients with mutated *IGHV* and no *TP53* aberrations. With this narrow indication, a decrease in the frequency of persistent cytopenias is predicted.

## Introduction

Over the past decades, the number of treatment options for chronic lymphocytic leukemia (CLL) has broadened with several effective medicines. There is increasing evidence that modern targeted therapies improve progression-free survival over chemoimmunotherapy in CLL in almost every clinical setting. First-line application of ibrutinib has gained more positive experience, especially for patients with unmutated *IGHV*. However, most of the clinical trials were unable to show a overall survival advantage in first line. Due to this fact and the financial restrictions in many countries, chemoimmunotherapy is still a viable first-line treatment option for CLL. Patients carrying 17p deletion and/or *TP53* mutation are currently the only exceptions; for CLL patients with these adverse prognostic markers, ibrutinib is the medication of choice.

Between January 2011 and January 2015, the fludarabine, cyclophosphamide, and rituximab (FCR) combination was the most easily available, effective, and supported chemoimmunotherapy for CLL patients in Hungary. This was the first protocol with not only progression-free survival but also with overall survival benefit [1–3]. FCR protocol is frequently criticized for its toxicity, but at the same time, it was widely used in the absence of a similarly effective alternative regimen. The mean age of the patients involved in these studies was 57 and 61 years, although the mean age of CLL patients requiring treatment is about 72–74 years. Due to the treatment-related toxicity and available alternative protocols, FCR is recommended as first-line therapy for fit patients who are younger than 65 years of age, and no *TP53* deletion/mutation can be detected. Since the available treatment options were restricted in several countries, including Hungary, for a long period, the FCR protocol was applied extensively, beyond its current indication in accordance with the original publications. The significant number of side effects was associated with the higher mean age. Short-term cytopenia and febrile neutropenia following chemotherapy are common in hematology and can usually be treated successfully. Among the side effects, persistent cytopenia, which requires repeated hospitalization and can even be fatal, is of high priority. Due to these experiences, FCR treatment is often regarded as a protocol that should be rendered obsolete.

In our recent study, we present the data of 120 patients treated with FCR between 2011 and 2015 compared to a cohort of 14 patients treated between 2016 and 2019. The effectiveness of the treatment is demonstrated by the remission rate and the progression-free survival. Our primary goal was to examine the prevalence of long-term cytopenias depending on fitness.

### Patients and Methods

Between January 2011 and January 2015, 120 CLL patients were treated with FCR. Data were collected using the electronic clinical records. To determine their suitability for the FCR treatment retrospectively, the age at the initiation of the treatment, kidney function, and comorbidity were taken into consideration. Recent recommendations regarding FCR treatment were not in use at the time of the study, so physicians used their judgment to establish fitness. For standardization of the comorbidities, the use of the Cumulative Illness Rating Scale (CIRS) is recommended by the German CLL study group (GCLLSG) with the consideration that its use is limited since it was originally developed for assessing physical impairment in the general geriatric population. CIRS value was obtained retrospectively from the comorbidity data available in the documentation. Another smaller cohort of 14 patients was added to the study as a comparison. They were treated between February 2016 and January 2019 when ibrutinib became available with broadening indications (*TP53* aberration in every treatment line, second line without restriction, and lately first line for *IGHV* unmutated patients).

FCR treatment was originally administered intravenously for 3 days in 28-days cycles. With the availability of the oral fludarabine, the adapted oral version of FCR was used exclusively by our center. Our preferred oral FCR combination consisted of fludarabine 40 mg/m^2^ for 3 days, cyclophosphamide 250 mg/m^2^ for 3 days and rituximab 375 mg/m^2^ in cycle 1, then 500 mg/m^2^ in cycles 2–6. An alternative combination was given for 5 days with fludarabine 25 mg/m^2^, cyclophosphamide 150 mg/m^2^ and rituximab 375 mg/m^2^ in cycle 1, then 500 mg/m^2^ in cycles 2–6. Patients unsuitable for full-dose FCR treatment could be treated with several dose-reduced regimes [4–6]. When the patient's creatinine clearance is less than 70 ml/min, it is recommended to reduce the dose of fludarabine by at least 50%. Instead of predefined FCR dose reductions, occasionally, the dosage of the drugs was individually modified by the physician according to age, kidney function, and documented or anticipated myelosuppression.

Response to treatment was assessed based on the blood test and physical examination according to the iwCLL 2008 recommendation. Since the time to next treatment is an easy-to-define, precise endpoint outside of clinical trials compared to the loss of response, the former was taken into account. Time to next treatment can be accurately determined using retrospective data, and it is also more relevant.

Cytopenia was considered to be persistent when it lasted for more than 2 months. Only grade 3–4 cytopenias were taken into account that persisted after the end of therapy and were treatment-related (neutrophil count lower than 1 G/L, hemoglobin level lower than 80 g/L, and platelet count lower than 50 G/L, according to the CTCAE V5.0 grading).

Pearson’s chi-squared test was used to compare categorical variables and assess independence.

The feasibility of the treatment is reflected by the number of the administered cycles and dose reductions. Dose reduction was the attending physician's responsibility, but the institutional recommendation was not to exceed 30% of the initially calculated dose. Complications occurring between treatment cycles and causing a delay in treatment will not be reported here.

## Results

Out of the 120 patients involved between 2011 and 2015, 86 received the FCR treatment in first line and 34 patients in second line or later. All the 14 patients received their treatment first-line between 2016 and 2019.

**FIGURE 1:**
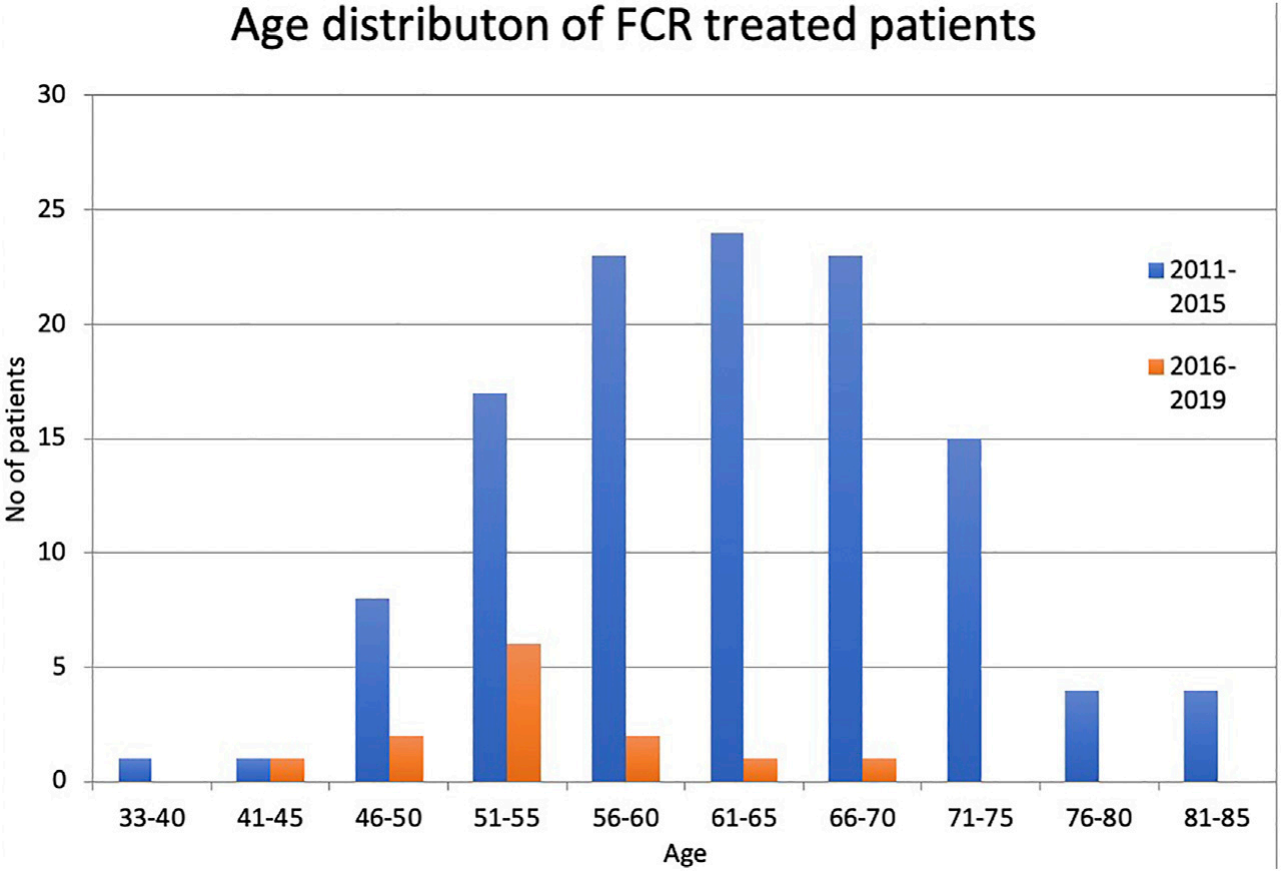
Age distribution of patients between 2011–2015 and 2016–2019.

The number of patients under 65 years of age was 74, 23 patients were between the age of 65–70, and 23 patients were older than 70 in the first cohort. This rate was similar for patients treated in first line and subsequent lines. The average age was 59 years ranging from 33 to 85. In the latter cohort, the average age was 53.5 years ranging from 45 to 66 (Figure 1). The dependence of fludarabine toxicity on kidney function is well established. Only 82 of the 120 patients (68.3%) had creatinine clearance 70 ml/min or above. Although all the patients with impaired kidney function received dose-reduced fludarabine, none of the dose reductions reached the recommended 50%. Fifteen patients had a CIRS score higher than 6, calculated retrospectively from the documentation.

According to today’s standards, a total of 45 patients, only 37.5%, would have been suitable to receive full-dose FCR from the first cohort. This distribution was similar in first-line and non-first-line therapy. Dose reduction was not applied in any of these patients, although 14 patients did not receive the planned 6 cycles. So, only 68.8% of the 45 patients who were potentially suitable for the treatment received 6 cycles of full-dose FCR. Dose reduction was not applied; reaching CR earlier and not the toxicity was the reason for reducing the number of cycles in all the 14 cases.

In addition, for 26 out of the 75 unfit patients, full-dose FCR treatment had been planned as well. Out of them, only 13 received the planned treatment (50%). The number of cycles was reduced for 6 patients, and the dose was reduced for 7 patients due to cytopenia.

For the 49 non-fit patients who received reduced-dose FCR from the beginning, 10 additional dose reductions were necessary. Only 27 patients, 55% of the 49 non-fit ones received the planned 6 cycles without further dose reduction.

**FIGURE 2:**
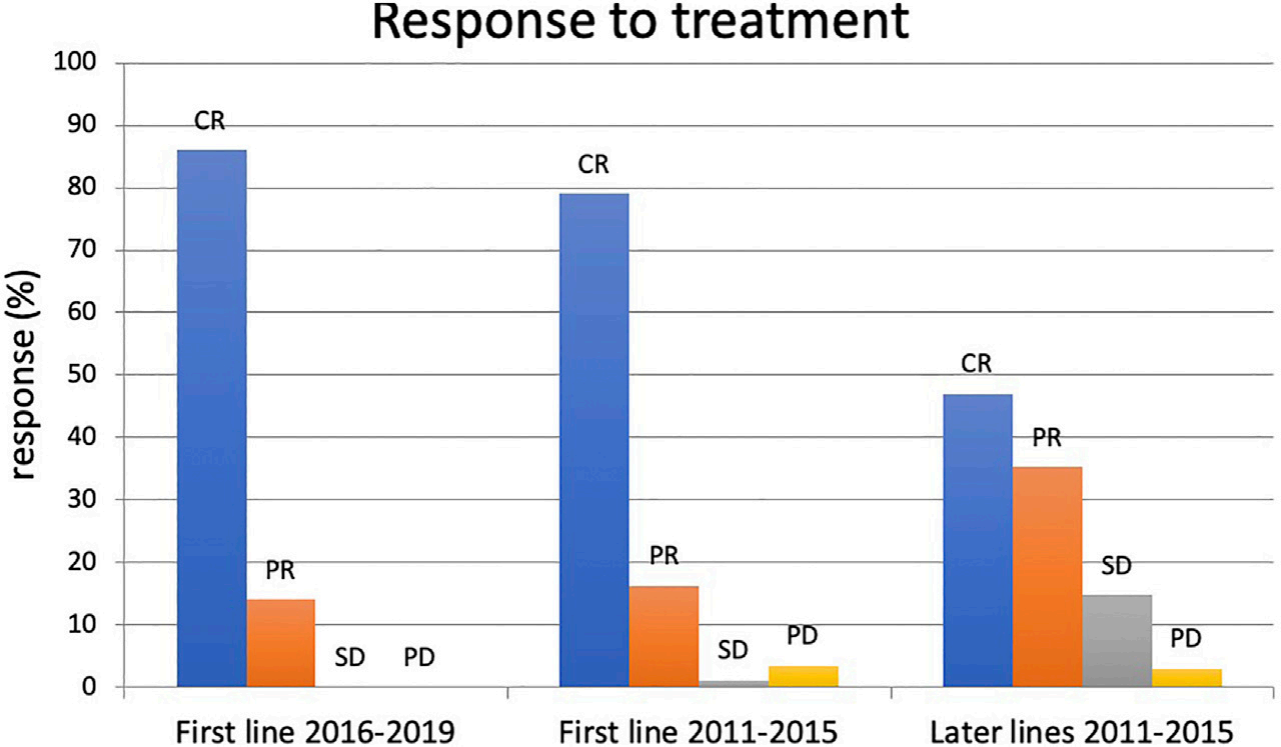
Result of FCR treatment between 2016–2019 and 2011–2015. CR: complete remission, PR: partial remission, SD: stable disease, PD: progressive disease.

Out of the 86 patients treated in first line, 68 had complete hematologic remission (CR 79.07%), 14 had partial remission (PR 16.28%), and 4 patients had stable or progressive disease (SD + PD 4.65%). This rate was significantly worse for those treated in non-first line (CR 47.05%, PR 35.29%, SD + PD 17.64%) (Figure 2).

In the 2016–2019 cohort, the complete remission rate was 86%, and all the remaining 14% achieved partial remission. One patient stopped treatment after 3 cycles due to hematological toxicity. No dose reduction occurred.

Table 1 illustrates the results achieved, regardless of the fitness calculated subsequently. Reducing the number of cycles and the dose shows a deviation from the original therapeutic plan. Both dose intensity modifications led to a significant decrease in the rate of complete remissions (*χ* ^2^(2) = 6.429, *p* = 0.04 for any dose modification, *χ* ^2^(2) = 6.824, *p* = 0.033 for lower cycle numbers).

**TABLE 1:** Response of patient treated with FCR in first line or in subsequent lines.

	Patient No	CR *n* (%)	PR *n* (%)	SD + PD (%)
First line treatment	86	68 (79.07%)	14 (16.28%)	4 (4.65%)
No dose reduction	45	36 (83%)	6 (15%)	1 (2%)
Cycles <6	28	19 (68%)	6 (21%)	3 (11%)
Dose reduction	13	10 (77%)	2 (15%)	1 (8%)
Subsequent lines of treatment	34	16 (47%)	12 (35%)	6 (18%)
No dose reduction	20	11 (55%)	8 (40%)	1 (5%)
Cycles <6	11	4 (36%)	3 (28%)	4 (36%)
Dose reduction	6	3 (50%)	2 (33%)	1 (17%)

CR: complete remission, PR: partial remission, SD: stable disease, PD: progressive disease.

**FIGURE 3:**
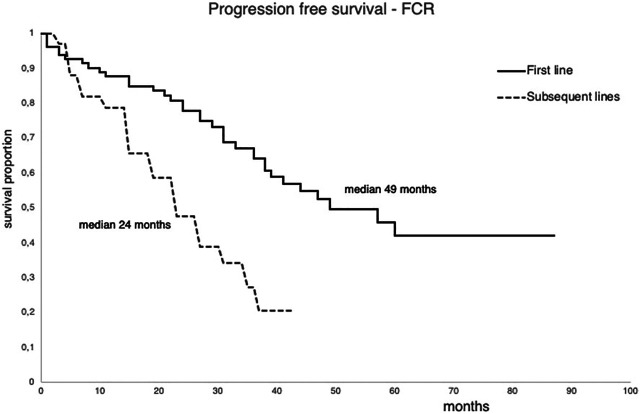
Progression free survival of the patient treated with FCR first line or in subsequent lines between 2011 and 2015.

The median time until the next treatment was 49 months for patients treated in first-line. Those patients who were treated in subsequent lines required treatment much earlier, in an average of 24 months (Figure 3).

### Long Term Cytopenias

Persistent cytopenia was observed in 17 cases (14%). In the three most serious cases requiring multiple hospitalizations, anemia was the primary reason, and it was associated with neutropenia in one case, and with thrombocytopenia in another one. The latter patient was the only one who died during persistent cytopenia; his cytopenia was observed for 7 months. Neutropenia was not observed during this period, but finally, sepsis was recorded as the cause of death. In other 14 cases, neutropenia lasting 3–8 months was observed, which caused febrile neutropenia in 10 cases; however, only one hospitalization was necessary. Among the patients suitable for full-dose chemotherapy, four had persistent cytopenia, and only two received FCR treatment in first-line. The proportion of the persistent cytopenia was the highest among those non-fit patients who were planned to receive a full-dose FCR treatment (7/26, 27%). When the patients received the dose suitable for their fitness, the proportion of persistent cytopenia was 10%. Furthermore, among fit patients treated in first line, the occurrence of persistent cytopenia was 4.6%.

In the later cohort, when ibrutinib was available as an excellent second-line choice and also as a first-line treatment for patients with *TP53* aberration, FCR was only recommended for those patients who were not older than 65 years, without major comorbidities and with creatinine clearance not less than 70 ml/min. Only one long-term cytopenia was observed in this cohort, whose fitness level was not evaluated properly. The creatinine clearance of this patient was 57 ml/min, and his treatment was permanently terminated due to severe transfusion-dependent anemia lasting for 5 months. After 3 cycles, he maintained his MRD positive CR for 28 months, and there was still no sign of progression on his last visit. The absence of fitness significantly correlated with the occurrence of cytopenias in the total patient population (*χ* ^2^ (1) = 6.001, *p* = 0.014 for all patients (*χ* ^2^(1) = 5.705, *p* = 0.017 for patients treated in first line).

## Discussion

Chronic lymphocytic leukemia is a disease of the elderly. The mean age of the patients diagnosed is over 70 years, and patients with treatment requirements are even older. The treatment aims to achieve remission and prolong the disease-free period as well as survival. Preserving the quality of life is also of high importance.

Chemoimmunotherapy was the mainstay of treatment for many years. FCR has the advantage of prolonging progression-free survival as well as overall survival, and it was widely used in almost all patient population. The benefits of FCR came at the expense of increased toxicity. Richter’s transformation, secondary malignancies, and long-term cytopenias deserve special mention. The underlying mechanism of transformation is still an active research area. Direct toxic effect, therapy-related immunosuppression, and consequential EBV reactivation are all considered explanations [7]. The lower incidence of Richter’s transformation after FCR compared to FC (13.1% vs. 17.4%) may underscore the role of deeper remission obtained with FCR and also the importance of the CLL progression itself [3]. The prevalence among relapsed/refractory patients treated with novel agents seems to be comparable to historical controls after chemoimmunotherapy [8]. The frequency of solid tumors didn’t increase compared to the general population. Secondary hematological malignancies observed after FCR therapy included 1.6–2.8–4.6% MDS/AML cases during long-term follow-up [3, 9, 10].

Persistent cytopenia that lasts for more than 2 months can overshadow the efficacy of FCR treatment resulting in increased visit numbers, hospitalization due to infections, or even death. Many hematologists reckon this an unexpected and unpredictable side effect that refrains them from using FCR. In our study, persistent cytopenia was observed in 17 cases (14%), which is comparable to the basic FCR studies with 17% and 19% prolonged cytopenias [3, 9]. The majority of them were prolonged neutropenia, and only two patients needed hospitalization due to infections, which shows that commonly used colony-stimulating factors are effective measures to avoid severe complications.

Recently, the indication of FCR is confined to fit and young patients. Chemoimmunotherapy is not recommended for relapsed/refractory patients anymore. The studies that established FCR as an excellent first-line choice included patients who didn’t meet today’s criteria. Both trials included patients older than 65 years, and the desirable kidney function was 176 umol/l in the MD Anderson Cancer Center trial, which probably equaled creatinine clearance lower than 70 ml/min in multiple cases. Comorbidities were assessed with CIRS score in the CLL8 trial, and only performance status was recorded in the MD Anderson CC trial. The putative cause of prolonged cytopenia was reported to be age and the advanced Rai stage in the later trial, but age was only mentioned in the early report [1, 3, 9]. In our study, the presumptive factors that led to persistent cytopenia were age and creatinine clearance (7-7 patients). If unfit patients had not been treated with FCR at all, and used only in first line, persistent cytopenia would have been observed in 4.6% of the cases. In our later cohort between 2016 and 2019, we used this approach and no long-term cytopenia was observed; the only exception was the patient whose GFR was below 60 ml/min. Interestingly, two patients developed pure red cell aplasia where cyclosporine A treatment was successfully used with complete correction of anemia.

The effectiveness of first-line FCR treatment is indisputable. The overall response rate is between 90 and 95%, the complete remission rate (CR) is 44–72% [1, 2, 11]. We also found FCR treatment to be very effective. The complete remission rate of those treated in first line was 79%. It should be noted that in clinical studies, complete remission was confirmed by using CT and bone biopsy. The IWCLL 2008 Guidelines only recommend the use of these examinations in clinical trials. Although long-term disease-free survivors can be found in our patient population, these data cannot be compared to international data due to the shorter observation time and the absence of knowledge of the *IGHV* mutation status.

Dose reduction and delay between cycles can usually be explained with toxicity. We chose this option in 14.1% and 10.8% of the cases. The number of cycles was reduced in 33% of the patients. Of these patients, 50% were in complete remission upon discontinuation of treatment. Despite this fact, if we examine the entire cohort, the reduction of the dose rate is associated with a significant reduction of the remission rate (*χ* ^2^(2) = 6.429, *p* = 0.04 for any dose modification, *χ* ^2^(2) = 6.824, *p* = 0.033 for lower cycle numbers, Table 1). This was observed in a few other trials, where different dose-reduced FCR regimes were administered to decrease toxicity. Even recently, efficacy and safety results with low-dose FCR in the elderly CLL population were published [12].

The number of FCR-treated patients significantly decreased from January 2016 when ibrutinib became available in our country. Although initially, first-line ibrutinib was restricted to patients with *TP53* aberration, its effectivity in second line probably changed the first-line treatment strategy, too, accepting less effective alternative chemoimmunotherapies easily. Based on the controlled studies available, the recommended first-line chemoimmunotherapy for clearly unfit and comorbid or elderly patients was the combination of anti-CD20 antibodies and chlorambucil. Out of the anti-CD20 antibodies, the obinutuzumab that provides the longest progression-free survival [13]. Where the obinutuzumab is of limited access, mostly rituximab + chlorambucil is used [14, 15]. A significant number of patients is in between the clearly unfit and fit states. For them, a treatment with rituximab + bendamustine (BR) was recommended [16, 17]. In the first period we examined, BR was not an easily available option either, so we usually used FCR.

We found that FCR treatment was feasible for a wide range of CLL patients. When the importance of long-term toxicity was noticed, dose reduction has become widely practiced after an occurrence of transient cytopenia. Decreasing the number of cycles can also reduce persistent cytopenias. FCR treatment of non-fit patients is not recommended anymore since the proportion of persistent cytopenias was the highest among them.

## Conclusions, the Future of FCR Treatment

The tremendous progress that has been made in the treatment of CLL will significantly affect the future use of chemoimmunotherapies like FCR. The past decade has seen the approval of several small molecules, including inhibitors of the BCL-2 and the B-cell receptor pathway with expanding indications. Duvelisib, a new phosphoinositide 3-kinase inhibitor, and acalabrutinib, another Bruton’s kinase inhibitor was recently added to the CLL treatment landscape, which is dominated by obinutuzumab, venetoclax and ibrutinib nowadays. Several phase 3 trials are testing first-line combinations of these new drugs aiming to achieve long-term efficacy together with limited toxicity. Therapies with fixed duration and MRD driven strategies are the main pillars of this approach. The results of clinical studies and not least, patient expectations will probably result in the exclusivity of chemotherapy-free regimes in the future.

Today, FCR treatment can still be recommended in first line for those patients who are younger than 65 years, without greater comorbidity, have good kidney function, and no *TP53* aberration. The “survival” of FCR can be substantiated by the data derived from the follow-up of patients involved in initial FCR studies. A few years ago, Keating et al. published the long-term data of their 300 patients, who were followed for an average of 12.8 years. At this time point, the PFS was 53.9% for those whose disease had a good prognosis based on the mutational status of the immunoglobulin heavy chain variable (*IGHV*) region, i.e., it was mutated (*IGHV*-M). Furthermore, 50.7% of patients with *IGHV*-M CLL were negative for minimal residual disease (MRD) after treatment resulting in further improvement in the PFS for this subgroup of patients, 79.8% at 12.8 years follow-up. There was a plateau on the PFS curve, which was unimaginable in previous CLL studies. The last relapse observed occurred at 10.4 years. No relapse was seen during the following 2.5 years of average observation period in 42 patients [10]. These patients are likely to be cured. The same conclusion can be drawn from the CLL8 trial for the patients with mutated *IGHV* [3].

Targeted therapies have changed the treatment recommendation in the past years. Recently, the CLL14 trial that compared venetoclax combined with obinutuzumab to chlorambucil plus obinutuzumab showed excellent results, probably establishing venetoclax plus obinutuzumab as a new standard of care with a fixed duration upfront therapy for patients with underlying conditions [18, 19]. Based on the results of the Resonate-2 study, ibrutinib is also widely used upfront [20–22]. However, long-term survival benefits of the first-line ibrutinib treatment instead of sequential therapy have yet to be proven.

There is growing evidence supporting the advantage of first-line ibrutinib treatment in CLL with poor prognosis, characterized by 11q deletion, unmutated *IGHV*, and complex karyotype. An important study from the perspective of the FCR is the ECOG1912 trial, where the combination of ibrutinib + rituximab is compared to standard FCR in patients not older than 70 years. Patients with the 17p deletion were not eligible. After 45 months of observational period, the PFS was superior in the ibrutinib plus rituximab arm (89% vs. 71%). This difference originated mainly from the results of the patients with unmutated *IGHV* (89% vs. 65%). The PFS results of the patients with mutated *IGHV* were comparable (88% vs. 82%). To draw a proper conclusion from the overall survival data needs further observation.

In most countries, the availability of targeted therapies in first line is still limited. In clinical practice, FCR remains an important treatment option for younger patients without major comorbidities and *TP53* aberration as well as adequate kidney function. It’s especially challenging to outperform the excellent data of patients with mutated *IGHV*, or at least it will take time. About 25% of patients with CLL are under 65 years of age, therefore the rate of patients receiving potential FCR treatment is already low. Due to the widespread use of less toxic novel agents, it is important to determine who is the most likely to enjoy the long-term benefits of FCR, so, today, the examination of the *IGHV* mutation status is compulsory according to the current iwCLL guideline [23]. Consequently, the number of patients receiving FCR treatment will continue to decrease.

Only rare cases of persistent cytopenias observed after FCR therapy can be described as unpredictable. However, it may be concluded that most of the prolonged cytopenias can be avoided by examining age, creatinine clearance, and comorbidity.

## Data Availability

The original contributions presented in the study are included in the article/Supplementary Material, further inquiries can be directed to the corresponding author.
